# Non-targeted metabonomics and transcriptomics revealed the mechanism of mulberry branch extracts promoting the growth of *Sanghuangporus vaninii* mycelium

**DOI:** 10.3389/fmicb.2022.1024987

**Published:** 2022-10-06

**Authors:** Jinxi Huo, Yuqing Sun, Meiliang Pan, Huanyan Ma, Tianbao Lin, Zhiqiang Lv, Yougui Li, Shi Zhong

**Affiliations:** ^1^Zhejiang Academy of Agricultural Sciences, Institute of Sericultural and Tea, Hangzhou, China; ^2^Department of Agriculture and Rural Affairs, Zhejiang Provincial Center for Agricultural Technology Extension, Hangzhou, China

**Keywords:** *Sanghuangprous vaninii*, mulberry branch extracts, liquid culture, hydrolase-related gene, non-targeted metabonomics

## Abstract

*Sanghuangprous vaninii* is a wood-inhabiting fungus, and its mycelium and fruiting body show excellent medicinal values. Mulberry is one of the major hosts of *S. vaninii*, however, the mechanism of mulberry affecting the growth of *S. vaninii* has not been reported. In the present study, a mulberry-inhabiting strain of *S. vaninii* was selected to explore the effects of mulberry branch extracts (MBE) on the growth of the strain. Results showed that MBE could significantly promote the growth of *S. vaninii* mycelium at the concentration of 0.2 g/l. After 16 days of liquid culture, the dry weight of mycelium in 0.2 g/l MBE medium was higher by three times compared with that in the control. The non-targeted metabonomic analysis of the culture medium at different culture times and concentrations was conducted to find the key components in MBE that promoted the growth of *S. vaninii* mycelium. Under the different concentrations of MBE culture for 10 and 16 days, 22 shared differential metabolites were identified. Next, in accordance with the peak value trend of these metabolites, HPLC–MS and liquid culture validation, four components derived from MBE (i.e., scopoletin, kynurenic acid, 3,5-dihydroxybenzoic acid and 2,4-dihydroxybenzoic acid) could significantly increase the growth rate of mycelium at the concentration of 2 mg/l. Transcriptomic and qRT-PCR analyzes showed that MBE could upregulate hydrolase-related genes, such as *serine–glycine–asparaginate–histidine* (*SGNH*) *hydrolase*, *alpha-amylase*, *poly-beta-hydroxybutyrate* (*PHB*) *depolymerase*, *glycosyl hydrolase family 61*, *cerato-platanin protein* and *Fet3*, which might enhance the nutrient absorption ability of *S. vaninii*. Importantly, MBE could significantly increase the content of harmine, androstenedione and vesamicol, which have been reported to possess various medicinal effects. Results suggested that MBE could be an excellent additive for liquid culture of *S. vaninii* mycelium, and these hydrolase-related genes also provided candidate genes for improving the nutrient absorption capacity of *S. vaninii*.

## Introduction

*Sanghuangprous vaninii* (Ljub.) L.W. Zhou and Y.C. Dai., formerly named *Phellinus gilvus* (Schwein.) Pat, is an important wood-inhabiting fungus that has been widely utilised in traditional medicine in China and adjacent countries (Shen et al., 2021). The mycelium and fruiting body of *S. vaninii* show excellent medicinal values. The fruiting body of *S. vaninii* shows significantly inhibitive effects on tumour cells (Wan et al., 2020, 2022; He et al., 2021; Yu et al., 2021; Guo et al., 2022; Qiu et al., 2022). In our previous study, the protocatechualdehyde from fruiting body can induce cell cycle arrest and apoptosis in HT-29 colorectal cancer cells and B16-F10 melanoma cells (Zhong et al., 2016, 2020). 3,4-dihydroxybenzalacetone, hydroxycinnamic acid, phellibaumin D, interfungin B, phelligridimer A and inoscavin A isolated from fruiting body show effective inhibitive effects on hepatocellular carcinoma cells HepG2 (Huo et al., 2020). In addition to the anti-carcinogenesis activity of fruiting body, the mycelium of *S. vaninii* shows excellent medicinal values. For example, the basal diet containing 5 g/kg *S. vaninii* dried mycelium can markedly improve the growth and innate immunity in weaned piglets (Sun et al., 2020). Also, the ethanol extracts of *S. vaninii* mycelium can reverse the loss of dopaminergic neurons and neurovascular reduction in 1-methyl-4-phenyl-1,2,3,6-tetrahydropyridine-induced Parkinson’s disease zebrafish model (Li et al., 2022). Research suggested that the mycelium and fruiting body of *S. vaninii* have remarkable commercial values and that the liquid fermentation of mycelium is an important process for harvesting the mycelium and large-scale artificial cultivation of *S. vaninii*.

Numerous macrofungi, such as *Lentinus edodes* and *S. vaninii*, parasitise woody plants. The components of the host can significantly affect the growth and metabolites of these macrofungi. For example, Wu et al. (2019) found that hemicellulose and lignin, the major components of wood, can stimulate mycelial growth and polysaccharide biosynthesis in *L. edodes*. Lignin can promote the growth of *S. vaninii* mycelia in culture plate at the concentration of 0.06 g/l (Guo et al., 2021). *S. vaninii* is a wood-inhabiting fungus that parasitises mulberry (Huo et al., 2020) and poplar (Shen et al., 2021). The effects of host on the mycelium of *S. vaninii* are worth studying.

In the present study, a mulberry-inhabiting strain of *S. vaninii* was selected to explore the effects of MBE on mycelial growth of *S. vaninii* by PDA plate culture and liquid fermentation. Next, the non-targeted metabonomic analysis of culture media was conducted to identify key components in MBE that might affect the growth of *S. vaninii* mycelium. Finally, the transcriptomic and metabonomic analyzes of mycelia were conducted to explore the mechanism of MBE affecting mycelial growth and production of active ingredients. Results will systematically evaluate the effects of MBE on the mycelium of *S. vaninii* and deepen our understanding of the interaction between host and inhabiting macrofungi.

## Materials and methods

### Strain culture and preparation of MBE

The strain of *S. vaninii* S12 (Huo et al., 2020) was isolated from the fruiting body grown in a mulberry tree in Tonglu, Zhejiang province of China (29.80° N, 119.67° E). A patch of the fruiting body was inoculated into potato dextrose agar (PDA) at 28°C. Mycelium free from contamination was stored at Institute of Sericulture and Tea, Zhejiang Academy of Agricultural Sciences, and the strain was ready for use after 7 days of culture on PDA at 28°C. In liquid culture, five colonies with size of 8 mm were punched from the PDA and added into 300 ml potato dextrose broth (PDB) in 500 ml flask. The mycelium was cultured at 200 rpm and 28°C, filtered to remove the culture medium and dried in an oven at 50°C for 2 days to obtain dried mycelium. All analytically pure reagents were purchased from Aladdin Co., Ltd. (China).

The dried mulberry branches were extracted with boiling water (w/v = 1:10) for 2 h. The filtered aqueous extracts were added with absolute ethanol at a ratio of 1:3. The supernatant after centrifugation was concentrated to 1/5 volume by rotary evaporator (30 rpm, 50°C, R502, Shensheng, China) and lyophilised by vacuum freeze drier (−50°C, 15 Pa; Alpha 1–4, Christ, Germany) to obtain MBE powders. The dried powders were stored at −20°C before use.

### Non-targeted metabonomic analysis

The non-targeted metabonomic analysis was conducted by Guangzhou Genedenovo Biotechnology Co., Ltd. (China). The mycelia (M) and culture media (CM) at different sampling times were collected. Mycelia (100 mg) were washed thrice by PBS buffer and ground with liquid nitrogen, and the homogenate was resuspended with prechilled 80% (v/v) methanol and 0.1% (v/v) formic acid by well vortex. CM (1 ml) were freeze-dried and resuspended with prechilled 80% (v/v) methanol and 0.1% (v/v) formic acid by well vortex. Samples were incubated on ice for 5 min and centrifuged at 15000 g and 4°C for 15 min. A certain amount of the supernatant was diluted to final concentration containing 53% (v/v) methanol by LC–MS-grade water. Samples were centrifuged at 15000 g and 4°C for 15 min. Finally, the supernatant was injected into the HPLC-MS/MS system (Want et al., 2006, 2013; Barri and Dragsted, 2013).

HPLC-MS/MS analyzes were performed using the Vanquish UHPLC system (ThermoFisher, Germany) coupled with the Orbitrap Q ExactiveTMHF-X mass spectrometer (ThermoFisher, Germany) in Guangzhou Gene Denovo Co., Ltd. (China). Samples were injected onto the Hypesil Gold column (100 × 2.1 mm, 1.9 μm) by using a 17 min linear gradient at a flow rate of 0.2 ml/min. The eluents for the positive polarity mode were eluents A (0.1% FA in water, v/v) and B (methanol). The eluents for the negative polarity mode were eluents C (5 mm ammonium acetate, pH 9.0) and D (methanol). The solvent gradient was set as follows: 2% B, 1.5 min; 2–100% B, 12.0 min; 100% B, 14.0 min; 100–2% B, 14.1 min and 2% B, 17 min. The Q ExactiveTM HF-X mass spectrometer was operated in positive/negative polarity mode with spray voltage of 3.2 kV, capillary temperature of 320°C, sheath gas flow rate of 40 arb and aux gas flow rate of 10 arb.

The raw data files generated by UHPLC–MS/MS were processed using the Compound Discoverer 3.1 (Thermo Fisher, Germany) to perform peak alignment, peak picking and quantitation for each metabolite. The main parameters were set as follows: retention time tolerance, 0.2 min; actual mass tolerance, 5 ppm; signal intensity tolerance, 30%; signal/noise ratio, 3 and minimum intensity, 100,000. After that, peak intensities were normalised to the total spectral intensity. Normalised data were used to predict the molecular formula based on additive ions, molecular ion peaks and fragment ions. Peaks were matched with the mzCloud,^1^ mzVault and Masslist database to obtain accurate qualitative and relative quantitative results. Three biological repeats were established at each sampling time. The VIP value of the orthogonal partial least squares discriminant analysis and P value of *t*-test were used to screen significantly different metabolites between different comparison groups, and the threshold of significant difference was as follows: VIP ≥ 1 and *t*-test *p* < 0.05 (Worley and Powers, 2013; Saccenti et al., 2014).

### Transcriptomic analysis

Mycelia at different sampling times were collected, and each sample had three biological replicates. Total RNA was isolated and purified using the TRIzol reagent (Invitrogen, United States) following the manufacturer’s instructions. RNA integrity, purity and concentration were assessed using the 2,100 Bioanalyzer (Agilent, United States), NanoPhotometer spectrophotometer (Implen, Germany) and Qubit 2.0 fluorometer (Invitrogen, United States), respectively. The construction of libraries and the RNA-Seq on the Illumina sequencing platform were performed by Guangzhou Genedenovo Biotechnology Co., Ltd. Raw reads were trimmed to remove adaptors and enhance quality by fastp (version 0.18.0, Chen et al., 2018). Parameters removed reads containing adapters, more than 10% of unknown nucleotides and low quality reads containing more than 50% of low quality (*Q*-value ≤20) bases. The HISTAT2.2.4 was used to map clean reads to the genome with default parameters (Kim et al., 2015). The StringTie v1.3.1 was used to assemble transcripts with mapped reads (Pertea et al., 2015). FPKM (Fragments Per Kilobase of transcript per Million fragments mapped) was used to measure transcript or gene expression levels (Florea et al., 2013). The predicted gene sequences were annotated functionally by COG, KEGG, swiss-prot and Nr databases (Tatusov et al., 2000; Boeckmann et al., 2003; Kanehisa et al., 2004; Deng et al., 2006). During the identification of differentially expressed genes, fold change (FC) ≥ 2 and false discovery rate (FDR) < 0.05 were used as screening criteria. Pearson correlation coefficients were calculated for metabolome and transcriptome data integration. Gene and metabolite pairs were ranked in descending order of absolute correlation coefficients. The top 250 pairs of genes and metabolites (with absolute Pearson correlation >0.5) were applied for metabolite–transcript network analysis by using igraph packages in R project (Csardi and Nepusz, 2006).

### Quantitative real-time PCR (qRT-PCR) analysis

Total RNA was isolated from mycelium at different sampling times. The PrimeScript RT reagent kit with gDNA Eraser (Takara Bio, Inc., Japan) and SYBR® Fast qPCR Mix (Takara Bio, Inc., Japan) were used for the CFX96 real-time PCR system (Bio-Rad Laboratories, Inc., United States). All operations were performed in accordance with the manufacturer’s instructions. The thermocycling conditions consisted of initial denaturation at 95°C for 30 s followed by 40 cycles at 95°C for 5 s and 60°C for 30 s. β-Actin was used as internal reference gene, and gene expression was quantified using the comparative 2^−ΔΔ^Cq method (Schmittgen and Livak, 2008). PCR primer sequences are listed in Supplementary Table 1.

### Statistical analysis

Data were expressed as mean ± SD. Statistical analysis was performed using the SPSS 16.0 software (SPSS, Inc.). One-way ANOVA was used to analyze statistical differences between groups under different conditions followed by Tukey’s *post-hoc* test. *p* < 0.05 indicated a significant difference.

## Results

### MBE could promote the growth of *Sanghuangporus vaninii* mycelium

**Figure 1:**
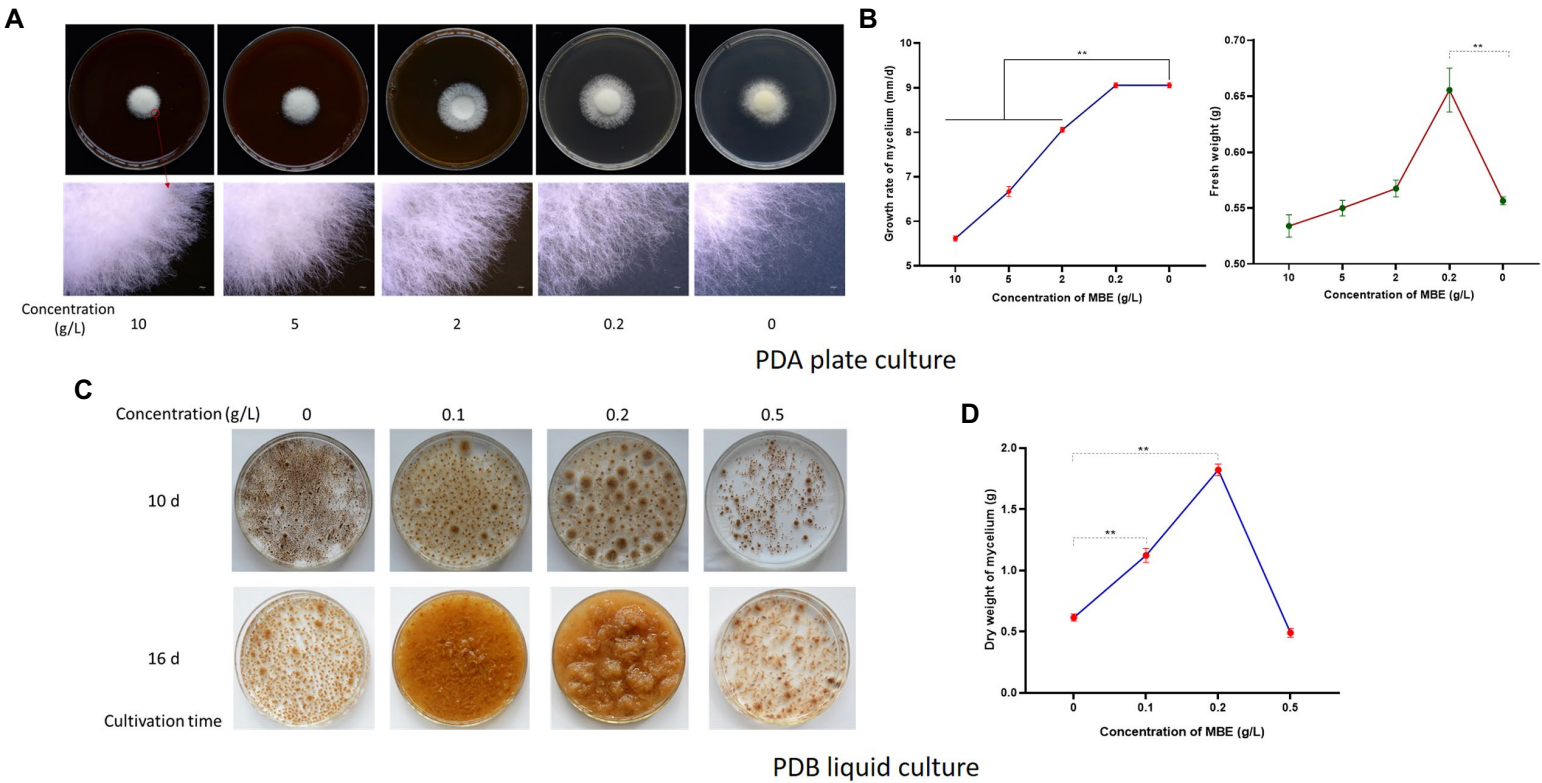
Effects of mulberry branch extracts (MBE) on *Sanghuangprous vaninii* mycelium. **(A)** Effects of different concentrations of MBE on the growth of *S. vaninii* mycelium on plate after 7 days of cultivation. The upper figures are the outline of the colony, and the lower figures are the 20× edge of colony. **(B)** Statistics of growth rate and fresh weight of *S. vaninii* mycelium on plate. **(C)** Effect of different concentrations of MBE on the growth of *S. vaninii* mycelium in liquid culture. **(D)** Statistics of dry weight of *S. vaninii* mycelium after 16 days of liquid cultivation. Three biological replicates were analyzed. Data are shown as means ± SEM. **p < 0.01.

The effects of different concentrations of MBE on the growth of *S. vaninii* mycelium were observed. As shown in Figures 1A,B, high concentrations (1 and 0.5 g/l) of MBE inhibited the expansion of *S. vaninii* mycelium, and the mycelium became dense. At the concentration of 0.2 g/l, MBE did not inhibit the expansion of mycelium and could significantly increase the fresh weight of mycelium on PDA plate (Figure 1B). Next, whether MBE could promote the growth of *S. vaninii* mycelium in liquid culture were observed in CM containing different MBE concentrations (0.5, 0.2 and 0.1 g/l). As shown in Figures 1C,D, 0.1 and 0.2 g/l of MBE in PDB could markedly promote the mycelium growth rate. After 16 days of liquid culture, the dry weight of mycelium in MBE (0.2 g/l) medium reached 1.82 g per 300 ml, which was three times higher than that of the control group (PDB without MBE). Results suggested that at the concentration of 0.2 g/l, MBE could significantly promote the growth of *S. vaninii* mycelium.

### Metabonomic analysis identified key active compounds in MBE

**Figure 2:**
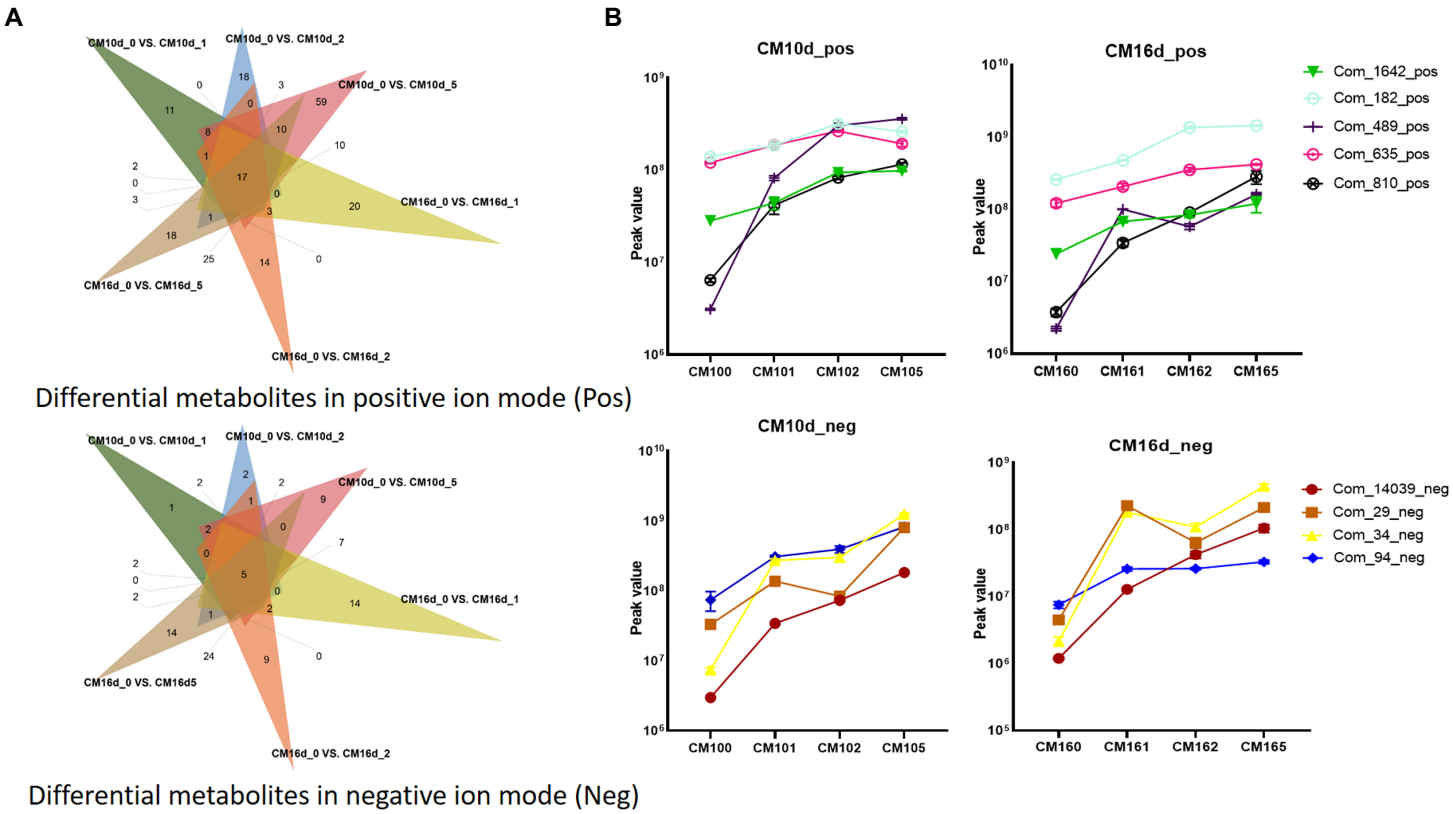
Metabolomic analysis of culture medium of *S. vaninii* mycelium. **(A)** Venn diagram of differential metabolites screening. **(B)** Peak value trend of screened metabolites.

The metabolomic analysis of the CM at different culture times (10 and 16 days) and concentrations (0.5, 0.2, 0.1 and 0 g/l) was conducted to find the key components in MBE that promoted the growth of *S. vaninii* mycelium. A total of 2,229 differential metabolites, including 1,509 in positive ion mode and 720 in negative ion mode, were identified. Under the different concentrations of MBE culture for 10 days (CM10d_1, CM10d_2 and CM10d_5) vs. control group culture for 10 days (CM10d_0), respectively, and CM16d_1, CM16d_2 and CM16d_5 vs. control group CM160, respectively. A total of 22 shared differential metabolites in the six groups above, including 17 in positive ion mode and 5 in negative ion mode (Figure 2A). Next, according to the peak value trend of different metabolites (Supplementary Figure 1), the peak values of groups with MBE should be higher than those of control groups (CM10d_0 and CM16d_0). Nine potential components, which might promote the growth of *S. vaninii* mycelium were screened (Table 1; Figure 2B). To confirm that these ingredients were derived from MBE and not mycelial secretion, the HPLC-MS analysis of aqueous MBE solution and standards of these reagents (N6-Succinyl Adenosine, polymer Trp-Met-His [WMH] and LDGTS 8:0 were unavailable) was conducted. As shown in Supplementary Table 2, all components except 4-hydroxycoumarin could be detected in aqueous MBE solution.

**Table 1:** Candidate components in MBE for promoting the growth of *Sanghuangprous vaninii* mycelium.

	Name		Formula	Molecular weight
*Positive ion mode (Pos)*				
Com_1642_pos	Trp-Met-His [WMH]		C_22_H_28_N_6_O_4_S	472.1919
Com_182_pos	N6-Succinyl Adenosine		C_14_H_17_N_5_O_8_	383.1076
Com_489_pos	Scopoletin		C_10_H_8_O_4_	192.0423
Com_635_pos	Kynurenic acid		C_10_H_7_NO_3_	189.0426
Com_810_pos	LDGTS 8:0		C_18_H_35_NO_6_	361.2464
*Negative ion mode (Neg)*				
Com_14039_neg	4-Hydroxycoumarin		C_9_H_6_O_3_	162.0317
Com_29_neg	3,5-Dihydroxybenzoic acid		C_7_H_6_O_4_	154.0266
Com_34_neg	2,4-Dihydroxybenzoic acid		C_7_H_6_O_4_	154.0265
Com_94_neg	Salicylic acid		C_7_H_6_O_3_	138.0316

**Figure 3:**
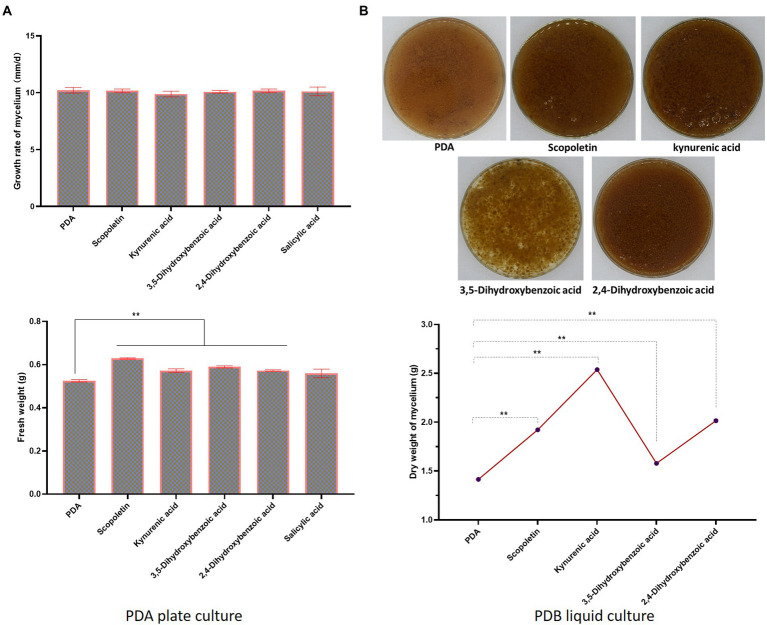
Effects of candidate metabolites in MBE on *S. vaninii* mycelium. **(A)** Statistics of growth rate and fresh weight of *S. vaninii* mycelium on plate. **(B)** Effects of four compounds on *S. vaninii* mycelium after 20 days of liquid cultivation. The upper figures are harvested mycelium, and the lower figures are the statistics of dry weight of *S. vaninii* mycelium after 20 days of liquid cultivation. Three biological replicates were analyzed. Data are shown as means ± SEM. **p < 0.01.

Next, the effects of the five components on promoting the growth of *S. vaninii* mycelium were verified. Three concentrations (20, 2 and 0.2 mg/l) were set to check the effects on growth rate and fresh weight of *S. vaninii* mycelium on PDA. At concentrations of 2 mg/l, scopoletin, kynurenic acid, 3,5-dihydroxybenzoic acid, 2,4-dihydroxybenzoic acid and salicylic acid showed better growth potential than other concentration gradients. However, the growth rate of mycelium showed no difference from that of PDA, but the fresh weight was significantly higher than PDA except salicylic acid (Figure 3A). Next, liquid culture was conducted to confirm whether scopoletin, kynurenic acid, 3,5-dihydroxybenzoic acid and 2,4-dihydroxybenzoic acid could increase the growth rate of mycelium. As shown in Figure 3B, after 20 days of liquid culture, these four components could significantly increase the dry weight of mycelium at concentrations of 2 mg/l. The medium containing kynurenic acid could harvest 2.54 g per 300 ml dried mycelium, which was 1.8-fold higher than that of the control.

### MBE primarily promoted the mycelial growth by upregulating hydrolase-related genes

**Figure 4:**
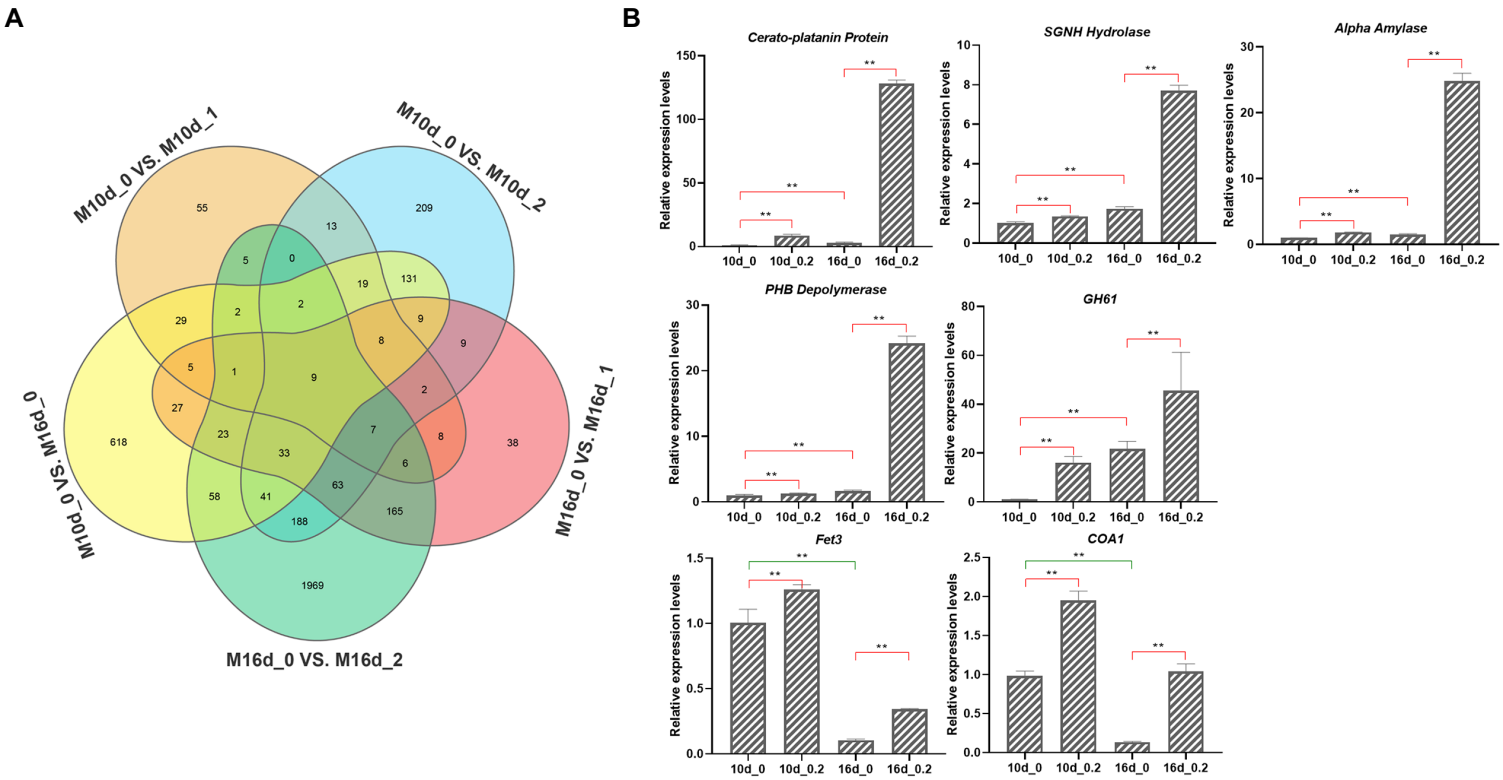
Transcriptomic analysis of *S. vaninii* mycelium. **(A)** Venn diagram of differential expression genes (DEGs). **(B)** qRT-PCR analysis of seven candidate genes. Four biological replicates were analyzed. Data are shown as means ± SEM. **p < 0.01.

To uncover the mechanism of promoted growth of *S. vaninii* mycelium by MBE, the transcriptomic analysis of mycelium, which was harvested at concentrations of 0, 0.1 and 0.2 g/l MBE for 10 and 16 days of liquid culture (M10d_0, M10d_1, M10d_2, M16d_0, M16d_1 and M16d_2) was conducted. According to the growth trend of mycelium, the parameters of screening differential expression genes (DEGs) were as follows: all upregulated DEGs in M10d_1 vs. M10d_0, M10d_2 vs. M10d_0, M16d_1 vs. M16d_0, M16 d_2 vs. M16 d_0 and M16 d_0 vs. M10 d_0 (Figure 4A). As shown in the Venn diagram (Figure 4A), the four groups involved in MBE could co-upregulate 16 genes, including 9 shared genes in M16 d_0 vs. M10 d_0 group (Figure 4A). Out of 16 genes, eight could be annotated, and two copies of *Fet3 protein* gene were annotated (Supplementary Table 3). Next, the relative expression levels of these seven genes by qRT-PCR at M10d_0, M10d_2, M16d_0 and M16d_2 were verified. All genes exerted higher expression levels at the concentration of 0.2 g/l than at the control at the same sampling time. The *Cerato-platanin protein*, *serine–glycine–asparaginate–histidine* (*SGNH*) *hydrolase*, *alpha-amylase*, *poly-beta-hydroxybutyrate* (*PHB*) *depolymerase* and *glycosyl hydrolase family 61* genes showed high expression levels at M16d_0 than M10d_0, whereas *Fet3* and *Cytochrome oxidase complex assembly protein 1* (*COA1*) were downregulated at M16d_0 compared with M10d_0 (Figure 4B). The *SGNH hydrolase*, *alpha-amylase*, *PHB depolymerase*, *glycosyl hydrolase family 61*, *cerato-platanin protein* and *Fet3* are all hydrolase-related genes. The proteins encoded by these genes could hydrolyse a variety of substrates. Thus, *S. vaninii* could absorb increased nutrients for mycelial growth.

### MBE significantly increased the content of active ingredients in the mycelium

The major application of liquid fermentation is the harvesting of mycelium. Therefore, the effects of MBE on mycelial components were analysed by metabolomics. The metabolomics analysis of M16d_2 vs. M16d_0 showed that 70 differential metabolites, including 53 in positive ion mode and 17 in negative ion mode, were identified. At M16d_2, 44 and 6 metabolites were significantly increased in the positive and negative ion modes, respectively, than at M16d_0 (Supplementary Table 4). Except those of some primary metabolites, the contents of active ingredients, such as harmine, androstenedione and vesamicol were significantly increased by more than 10-fold by MBE (0.2 g/l), whereas acetylcholine, N-acetylputrescine and caprolactam decreased significantly under MBE (0.2 g/l) treatment (Supplementary Table 4). Harmine has various pharmacological activities, such as anti-inflammatory and antitumor properties (Zhang et al., 2020). Androstenedione, a steroidal hormone, is thought to be an enhancer for athletic performance and build body muscles (Badawy et al., 2021). Vesamicol, a selective vesicular acetylcholine transporter inhibitor, and acetylcholine can antagonistically regulate cholinergic transmission to treat cholinergic dysfunction-associated disorders (Muramatsu et al., 2022). Results implied that the mycelium harvested from 0.02% MBE liquid culture might possess improved medicinal effects.

**Figure 5:**
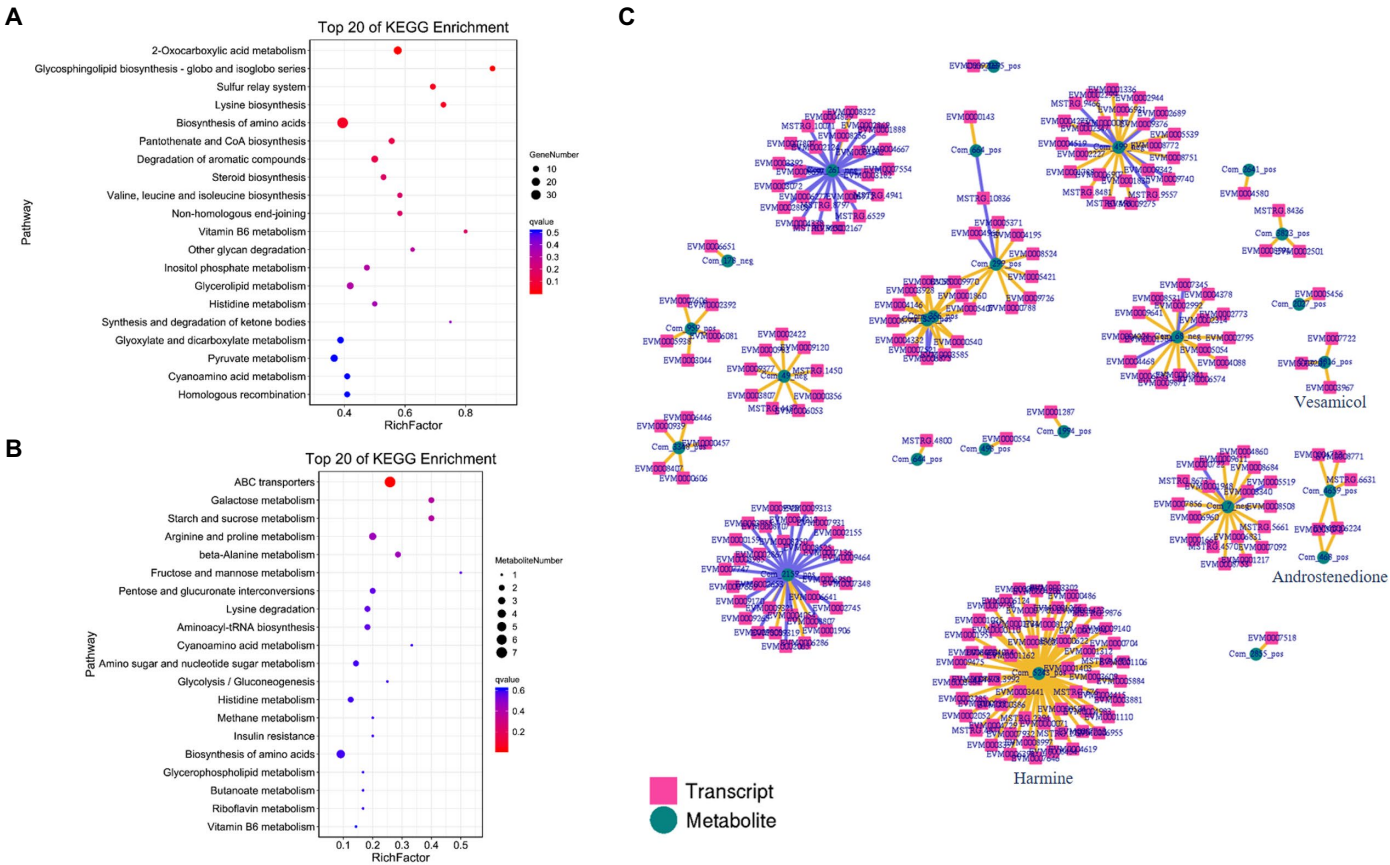
Correlation analysis between mycelial metabolites and transcriptomics (M16d_2 vs. M16d_0). **(A)** KEGG DEGs in M16d_2 vs. M16d_0. **(B)** KEGG differential metabolites in M16d_2 vs. M16d_0. **(C)** Pearson correlation coefficient calculation of DEGs and differential metabolites.

To further explore the synthesis mechanism of these active ingredients, correlation analysis between mycelial metabolites and transcriptomics (M16d_2 vs. M16d_0) was carried out. KEGG analysis showed that DEGs were enriched in the 2-oxocarboxylic acid metabolism pathway and biosynthesis of amino acids pathway (Figure 5A), and differential metabolites were primarily enriched in ABC transporter pathway (Figure 5B). These enriched pathways were mostly related to the growth difference of the mycelium rather than the biosynthesis of these active ingredients. Therefore, the Pearson correlation coefficient model was used to determine the relationship between DEGs and differential metabolites. As shown in Figure 5C, harmine showed strong positive correlation (>0.99) with 58 genes, and androstenedione and vesamicol correlated positively with 2 and 3 genes, respectively. In accordance with the description of these correlated genes (Supplementary Table 5), several hydrolase family genes were annotated. In addition, cytochrome p450 genes and genes of unknown function might be involved in regulating the biosynthesis of these active ingredients. The characteristics of these genes need further research.

## Discussion

As a valuable medicinal fungus, *S. vaninii* has the characteristics of slow growth and weak competitiveness, and the improvement of growth rate of mycelium can largely overcome these shortcomings. In the present study, the extracts from host of *S. vaninii* can significantly promote the growth of *S. vaninii* mycelium in liquid culture. Guo et al. (2021) found that the polymer lignin, as a component of branches, can promote the growth of *S. vaninii* mycelium at the concentration of 0.06 g/l. Findings imply that MBE may have similar effects. In the present study, after 16 days of liquid culture, the dry weight of mycelium in 0.2 g/l MBE medium reaches 1.82 g per 300 ml, which is thrice that of the control. Metabonomic analysis is conducted to identify key active compounds in MBE. Components derived from PDA are excluded by screening the differential shared metabolites of CM10d_0 vs. CM10d_1, CM10d_0 vs. CM10d_2, CM10d_0 vs. CM10d_5, CM16d_0 vs. CM16d_1, CM16d_0 vs. CM16d_2 and CM16d_0 vs. CM16d_5 (Figure 2A). Next, if the peak values of the groups with MBE are lower than the control groups (CM10d_0 and CM16d_0), these metabolites are thought be the reduced mycelial secretion under MBE treatment rather than components in MBE. And nine potential components are screened (Table 1). Further, five ingredients derived from MBE rather than mycelial secretion (Supplementary Table 2) are confirmed by HPLC-MS. And 4-hydroxycoumarin is derived from mycelial secretion. About 2 mg/l scopoletin, kynurenic acid, 3,5-dihydroxybenzoic acid and 2,4-dihydroxybenzoic acid can significantly increase the dry weight of mycelium in liquid culture, and the medium containing 2 mg/l kynurenic acid can harvest 2.54 g dried mycelium, which is 1.8-fold than that of the control group (Figure 3B). All four compounds are not reported to promote the growth of fungal mycelium. Unfortunately, none of these four compounds can achieve the same growth-promoting effects as MBE. Several components in MBE may work together to promote the growth of *S. vaninii* mycelium, and some minor effects of compounds have not been identified.

The enhancement of the vitality of *S. vaninii*, as a wood-inhabiting fungus, primarily depends on the improved activity of the degrading complex and recalcitrant plant polymers, secreting different enzymes that hydrolyse plant cell wall polysaccharides (Guerriero et al., 2015). Polysaccharides are embedded in plant cell walls and form a network of chains bound to cellulose and pectin. Matrix polysaccharides are structurally complex and are substituted by various carbohydrates and acids (Urbániková, 2021). Upregulated genes among M10d_1 vs. M10d_0, M10d_2 vs. M10d_0, M16d_1 vs. M16d_0, M16 d_2 vs. M16 d_0 and M16 d_0 vs. M10 d_0 were screened by transcriptomics and qTR-PCR analysis (Figure 4). A total of 6 of 7 annotated genes possessed hydrolase activity. *Cerato-platanin protein*, *SGNH hydrolase*, *Alpha amylase*, *PHB depolymerase*, and *Glycosyl hydrolase family 61* genes showed higher expression levels at M16d_0 than at M10d_0, whereas *Fet3* and *COA1* were downregulated at M16d_0 than at M10d_0 (Figure 4B). Majority of the upregulated genes belongs to hydrolase-related genes. SGNH hydrolase and PHB depolymerase belong to the carbohydrate esterase family, which can remove the *o*-acetylation modification of polysaccharides for the complete degradation of the plant cell walls. The glycosyl hydrolase family 61 can hydrolase carboxymethyl cellulose and β-glucan (Karlsson et al., 2001). Alpha-amylase, also called GH13, is responsible for the endohydrolysis of (1 → 4)-α-D-glucosidic linkages in polysaccharides (Janíčková and Janeček, 2021). Fet3, a kind of laccase, can degrade lignin and humus (Janusz et al., 2020). Some of cerato-platanin coding proteins are produced during infection by pathogenic fungi (Yu and Li, 2014). Li et al. (2019) characterised a cerato-platanin-like protein from *Fusarium oxysporum* named FocCP1. In tobacco, the FocCP1 protein can cause the accumulation of reactive oxygen species, formation of necrotic reaction, deposition of callose, expression of defence-related genes and accumulation of salicylic and jasmonic acids in tobacco. These hydrolase-related proteins can hydrolyse complex polysaccharides, including lignin, cellulose and callose. As a result, *S. vaninii* can absorb plenty of nutrients for mycelial growth. COA1 participates in the synthesis of phosphatidylinositol (PI). PI is an important secondary messenger that can affect diverse cellular processes, including protein transport, cell polarity, cytoskeletal organisation, ion-channel function and gene expression (Finkelstein et al., 2020). *Fet3* and *COA1* are downregulated at M16d_0 than at M10d_0 (Figure 4B), suggesting that *Fet3* and *COA1* can respond to MBE treatment at an early stage. The expression levels of five other genes are high at 16 days under MBE treatment, which may be responsible for further promoting the growth of *S. vaninii* mycelium.

Liquid fermentation can provide strains for large-scale artificial substitute cultivation and directly harvest mycelium for the extraction of bioactive compounds. MBE (0.2 g/l) can remarkably increase the contents of harmine, androstenedione and vesamicol by more than 10-fold (Supplementary Table 4). Harmine has various pharmacological activities, such as anti-inflammatory, antitumor, antidiabetic and neuroprotective activities. Moreover, harmine exhibits insecticidal, antiviral and antibacterial effects (Zhang et al., 2020). Previous study (Sun et al., 2020) found that *S. vaninii* mycelium can markedly improve growth and innate immunity in weaned piglets and that the mycelium harvested from MBE (0.2 g/l) liquid culture may possess improved effects. Androstenedione, a steroidal hormone, is thought to be an enhancer for athletic performance, build body muscles, reduce fats, increase energy, maintain healthy red blood cells and increase sexual performance (Badawy et al., 2021). No relevant medicinal effect of *S. vaninii* mycelium has been reported. In the MBE (0.2 g/l) group, vesamicol, a selective vesicular acetylcholine transporter inhibitor, increases, whereas acetylcholine decreases, implying that the mycelium in MBE (0.2 g/l) group can also treat cholinergic dysfunction-associated disorders. Pearson correlation analysis showed that harmine has strong positive correlation (>0.99) with 58 genes. Except several hydrolase family genes, majority of genes are cytochrome p450s and genes of unknown function (Supplementary Table 5), which may be involved in regulating the biosynthesis of these active ingredients. The medicinal efficacy and gene function of *S. vaninii* need further exploration.

## Conclusion

In the present study, MBE could promote the growth of *S. vaninii* mycelium at the concentration of 0.2 g/l and identified four key active compounds in MBE, which were primarily responsible for the growth-promoting effects. In addition, MBE promoted the mycelial growth by upregulating hydrolase-related genes. Finally, MBE could significantly increase several bioactive ingredients in mycelium. Results suggested that MBE is an excellent additive for the liquid culture of *S. vaninii* mycelium.

## Data availability statement

The raw RNA-seq data for this study can be found in the NCBI database – BioProject ID: PRJNA871986. The non-targeted metabonomic datasets can be found in Figshare – https://doi.org/10.6084/m9.figshare.20524158.v1.

## Author contributions

YL and SZ conceived and designed the study. JH performed the experiments with the help of YS, MP, HM, TL, and ZL. YL, SZ, and JH analyzed the data and prepared the manuscript. All authors contributed to the article and approved the submitted version.

## Funding

This work was supported financially by Science and Technology Department of Zhejiang Province (LQ21C150002 and 2018C02003).

## Conflict of interest

The authors declare that the research was conducted in the absence of any commercial or financial relationships that could be construed as a potential conflict of interest.

## Publisher’s note

All claims expressed in this article are solely those of the authors and do not necessarily represent those of their affiliated organizations, or those of the publisher, the editors and the reviewers. Any product that may be evaluated in this article, or claim that may be made by its manufacturer, is not guaranteed or endorsed by the publisher.

## Supplementary material

The Supplementary material for this article can be found online at: https://www.frontiersin.org/articles/10.3389/fmicb.2022.1024987/full#supplementary-material

## Footnotes

^1^ https://www.mzcloud.org/
