# Supplementary figures and images for: Non-targeted metabonomics and transcriptomics revealed the mechanism of mulberry branch extracts promoting the growth of Sanghuangporus vaninii mycelium
Source: Front Microbiol. 2022 Oct 6;13:1024987. doi: 10.3389/fmicb.2022.1024987 (PMC9582429; doi:10.3389/fmicb.2022.1024987)

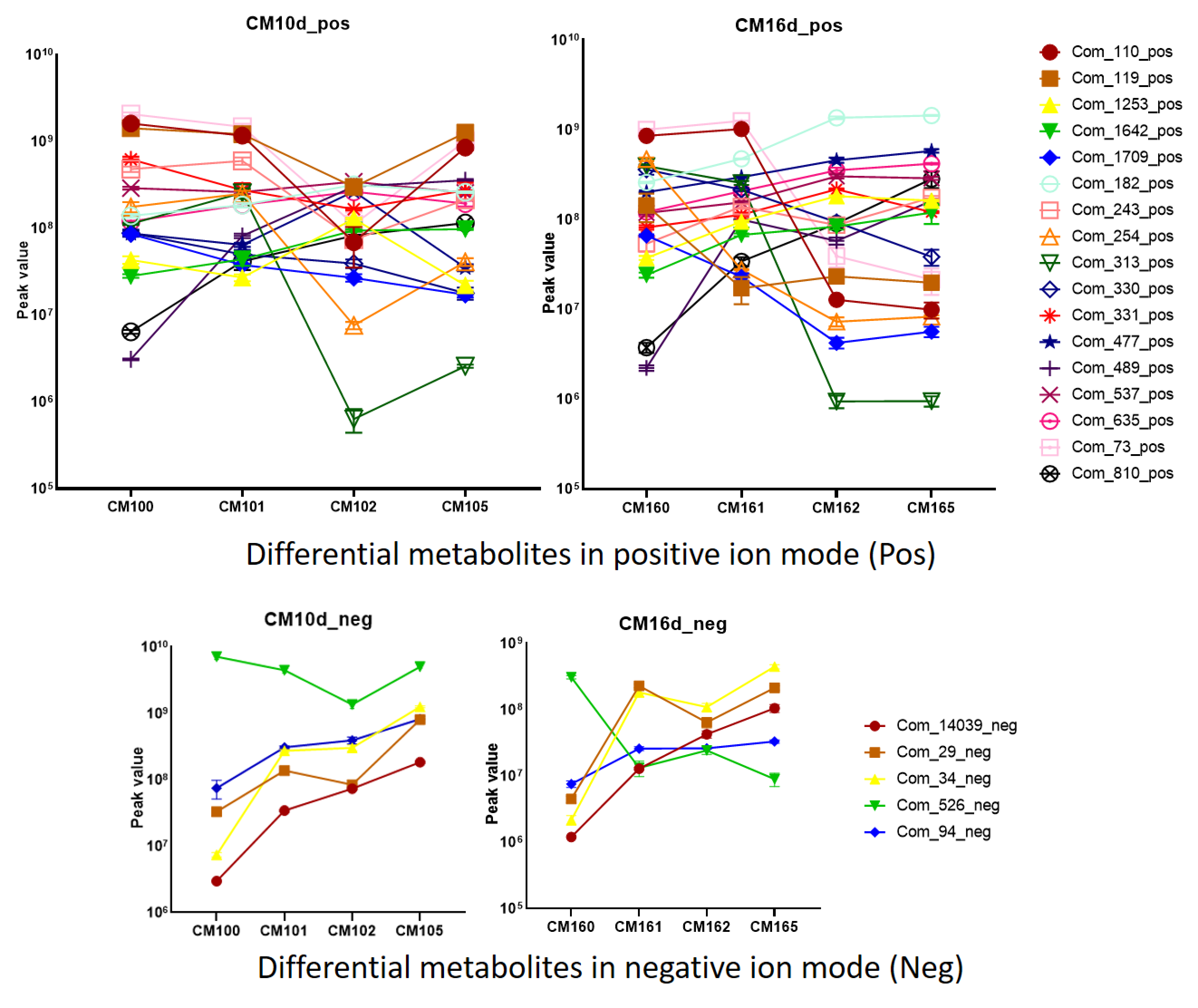


**Supplementary Figure 1** The peak value trend of 22 candidate metabolites.

Supplement: Supplementary file 2 [file Data_Sheet_2.docx]

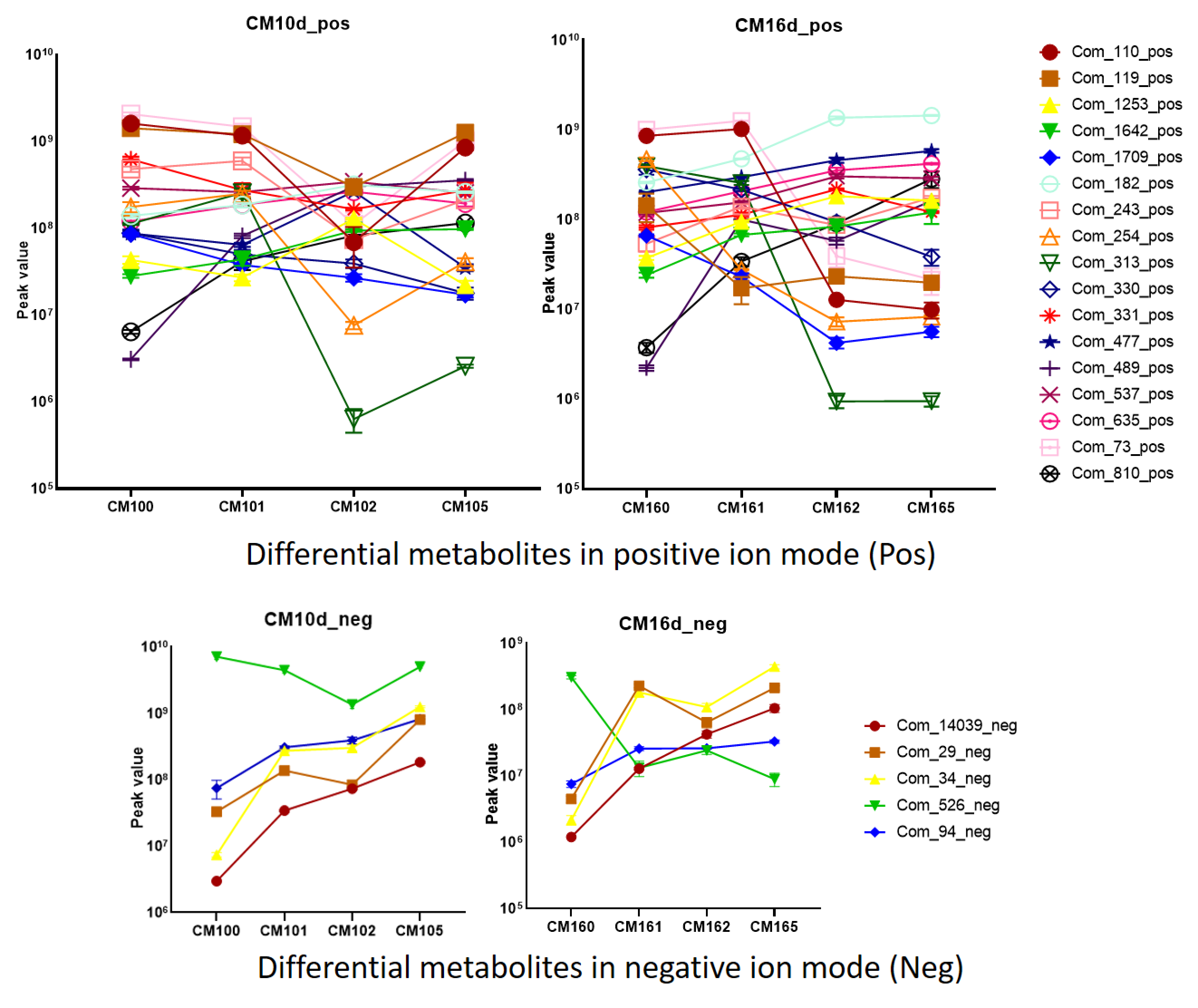

Supplement: Supplementary file 3 [file Image_1.TIF]
